# On the self-assembly of αB-crystallin

**DOI:** 10.1039/d5sm00684h

**Published:** 2025-08-25

**Authors:** Ewelina Lindbladh, Marija Dubackic, Dev Thacker, Sara Linse, Ulf Olsson

**Affiliations:** a Biochemistry and Structural Biology, Chemical Center, Lund University Lund Sweden ewelina.lindbladh@biochemistry.lu.se; b Physical Chemistry, Chemical Center, Lund University Lund Sweden majuskad@hotmail.com; c Astbury Centre for Structural Molecular Biology, University of Leeds Leeds UK

## Abstract

The molecular chaperone αB-crystallin is a small heat shock protein that inhibits the aggregation of, among others, Aβ42 and α-synuclein. These proteins are major hallmarks of Alzheimer's and Parkinson's disease, respectively. In order to understand the mechanism with which αB-crystallin performs its chaperone function it is essential to characterize its self-assembly in terms of aggregate size distribution, structure, and critical concentration. The size distribution of the assemblies has been widely discussed and they have been suggested to be monodisperse or polydisperse with varying size distributions covering a range of 10–40 monomers per assembly. Here, the size distribution was studied using dynamic and static light scattering, microfluidic diffusional sizing (MDS), as well as small-angle X-ray scattering (SAXS). Findings indicate that αB-crystallin has a preference toward forming spherical assemblies consisting of 18 monomers with a hydrodynamic radius of ≈7 nm after one week. SAXS data were modelled using a homogeneous sphere model with a radius of 6 nm, which is comparable to the light scattering and MDS results. 2D classes built from negative stain transmission electron microscopy images suggest that the spherical aggregates contain several smaller globular units. Furthermore, the findings show that the size of the assemblies is independent of protein concentration, supporting a strong preference for specific assembly constellations.

## Abstract 2

In this work the self-assembly of the chaperone αB-crystallin is shown to follow the closed association model to a good approximation. The oligomeric form is found to consist of 18 monomers, approximately 7 nm in radius, independent of concentration.

## Graphical abstract

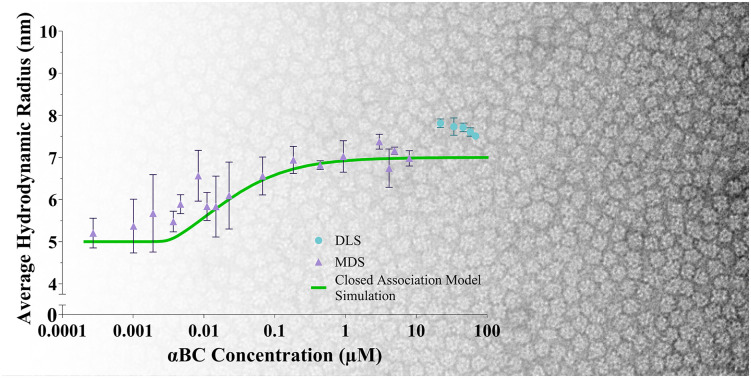

## Introduction

Chaperones are a class of proteins capable of maintaining other proteins – clients – in a functional state by preventing their misfolding and aggregation. Chaperones are often classified as foldases or holdases, where the former class assists in the folding of clients at the expense of ATP, and the latter class prevents misfolding and aggregation through intermolecular interactions without external energy input.^1^

One group of chaperones is the small heat shock protein family, which includes the crystallin proteins. These are found at high concentrations in the eye lens, where they maintain its transparency and high refractive index.^2,3^ There are three main types of crystallins: α-crystallin, β-crystallin, and γ-crystallin. α-Crystallin consists of two different polypeptide chains, αA-crystallin and αB-crystallin, that have been suggested to form co-assemblies in a 3 : 1 stoichiometry in the eye lens.^4^ While closely related in sequence, αA-crystallin is predominantly expressed in the eye lens while αB-crystallin (αBC) is ubiquitously expressed throughout the body, including the eye lens, the brain, as well as the heart and skeletal muscles (data available from https://v23.proteinatlas.org).^5–7^

The chaperone activity of αBC may be limited to a holdase function, preventing unfolded proteins from misfolding and aggregation. It has previously been shown that αBC aids in maintaining proteostasis in several systems related to neurodegenerative diseases through the suppression of the amyloid formation of β_L_-crystallin,^4^ lysozyme,^8,9^ β2-microglobulin,^8,10^ α-synuclein,^11–13^ Aβ40 and Aβ42,^10^ and insulin.^9^

**Fig. 1:**
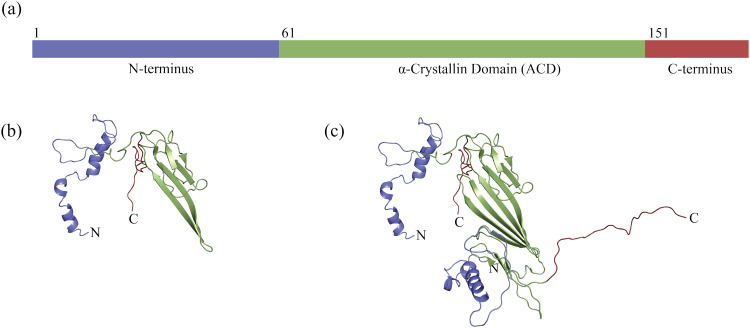
The regions and one proposed structure of αB-crystallin. The N-terminal region is colored blue, the α-crystallin domain is colored green, and the C-terminal region is colored red (PDB: 3J07).^16^ (a) Schematic of the αB-crystallin protein sequence. The N-terminal region consists of residues 1 to 60, the α-crystallin domain of residues 61–150, and the C-terminal region of residues 151–175.^15,16^ (b) The monomer structure in an oligomer of αBC consisting of 24 monomers. (c) The dimer structure in an oligomer of αBC consisting of 24 monomers.

The αBC protein, Fig. 1, consists of 175 amino acid residues that form an N-terminal region, rich in hydrophobic residues, an α-crystallin domain (residues 67–157,^14^ 61–150,^15,16^ or 69–150^17^), and a C-terminal region. The α-crystallin domain is a distinguishing feature of small heat shock proteins, and structural investigations by solid-state NMR spectroscopy and X-ray crystallography have revealed the domain to be dominated by β-sheet.^14,18^

The isolated α-crystallin domain has been found to associate into dimers but not higher order assemblies.^14^ A representation of the dimer can be viewed in Fig. 1(c), where the α-crystallin domains of two monomers form a joint, elongated, β-sheet in the αBC dimer. Based on inspection of the structural model, the dimer has been proposed to be stabilized by electrostatic and hydrophobic interactions between N- and C-termini.^19^ Furthermore, this dimer-state of αBC has been suggested to be the main building block for the assembly of higher-order aggregates in several studies^15,17,18,20–23^ and as reviewed by Dimauro *et al.*^17^ The dimer–dimer interface includes a well-conserved Ile–X–Ile motif (residues 159–161) located in the C-terminal region of a monomer in one dimer, which contacts a hydrophobic pocket of a monomer in a second dimer.^16,19^ The higher-order assemblies appear to be further stabilized by interactions between dimer N-termini.^19^

Within the assembly, hexameric rings consisting of three dimers can be identified.^17,24^ See Fig. 1(b) and (c) for the structural models of monomeric and dimeric αBC, built on experimental data from solid-state NMR spectroscopy, SAXS, and electron microscopy.^16^

The self-association of recombinant αB-crystallin, including its oligomer size distribution, assembly structure, and dynamics have previously been studied by, but not limited to, size exclusion chromatography (SEC),^25^ dynamic light scattering (DLS),^25,26^ mass spectrometry (MS),^21,22,27,28^ NMR spectroscopy,^19,20^ electron microscopy (EM),^9,19,21,26^ microfluidic high-field electrophoresis,^29^ and analytical ultracentrifugation (AUC).^19,25^ These studies report on size distributions as wide as approximately 10–40 monomers and are typically performed at a single protein concentration.^21,27^ Some studies report smaller ranges than 10–40 monomers per oligomer, however, they are all within that wider range.^26,30^ Despite the numerous studies on αB-crystallin, there is still no agreement on whether the size distribution of the oligomers is monodisperse or polydisperse in nature.

This study is motivated by the lack of consensus regarding the width of polydispersity of αB-crystallin rather than the average of monomers per oligomer in combination with the lack of characterization of its concentration dependence over a broad concentration range. To investigate this further, this work utilizes several scattering techniques (dynamic light scattering, DLS; static light scattering, SLS; and small-angle X-ray scattering, SAXS) and microfluidic diffusional sizing (MDS) and negative staining electron microscopy (EM) to systematically study the size, shape, and size distribution of recombinant αB-crystallin as a function of protein concentration and equilibration time. The data presented herein support that the distribution of αB-crystallin closely resembles a monodisperse size distribution.

## Materials and methods

### Protein production and purification

Recombinant human αB-crystallin was expressed in *E. coli* (BL21 DE3 pLysS star) from a synthetic gene with *E. coli*-optimized codons cloned in a Pet3a vector (Purchased from Genscript, Piscataway). Cells from 1 L culture were sonicated in 80 mL Tris buffer (10 mM Tris, 1 mM EDTA, pH 8.0) on ice followed by centrifugation for 15 min at 15 000 rpm at 4 °C (Beckman, SS25:50 rotor) whereafter the supernatant was collected. 17% of the material was used for purification, the remaining stored for future use. The 30–50% ammonium sulphate (AMS) fraction was obtained as follows: 13.3 mL supernatant was mixed with 5.7 mL saturated AMS, pH 8.0, incubated on ice for 10 min before centrifugation at 8500 rpm for 15 min at 4 °C (Biofuge). The supernatant was collected and supplemented with 7.6 mL saturated AMS, pH 8.0, incubated on ice for 10 min followed by centrifugation at 8500 rpm for 15 min. The pellet was collected, dissolved in 30 mL Tris-HCl buffer (10 mM Tris-HCl, 1 mM EDTA, pH 8.0) and again precipitated with 50% AMS and collected by centrifugation. The pellet was collected and dissolved in 500 mL Tris buffer (10 mM Tris, 1 mM EDTA, pH 8.0) and dialyzed against H_2_O overnight at 4 °C.

The dialyzed solution was pumped through a 5 mL QHP column and eluted using a linear NaCl gradient (0.0 M–0.2 M). Eluted fractions were further purified using two rounds of size exclusion chromatography (SEC) on a Superdex200 26/600 column, first in 20 mM sodium phosphate, 0.2 mM EDTA, 1.5 M GuHCl, pH 8.0, and then in 20 mM sodium phosphate, 0.2 mM EDTA, pH 8.0. Further information on the purification, including SDS-PAGE gels and chromatograms, can be found in Section S1 in the SI. The concentrations of the eluted fractions was determined by the absorbance at 280 nm (Labbot) assuming an extinction coefficient of 14 000 M^−1^ cm^−1^. The purified protein was stored at −20 °C.

### Fluorescent protein production and purification

An αB-crystallin mutant with a cysteine residue added directly after the initial methionine residue was expressed and purified in the same manner as described above, but with the inclusion of 1 mM dithiothreitol (DTT) in all buffers except during the final size exclusion chromatography (SEC) step. The protein obtained from this last step was mixed with 2 molar equivalents of AlexaFluor647-maleimide, added from a 5 mM stock in DMSO. The mixture was incubated for 2 h at room temperature after which it was lyophilized. The lyophilized sample was dissolved in 6 M GuHCl, incubated 1 h and again purified by SEC to remove excess dye and to obtain pure αBC-NCys-Alexa647, which was aliquoted and stored frozen.

All buffers were filtered through 0.2 μm filter paper (Pall Corporation, water wettable polytetrafluoroethylene (wwPTFE)) and degassed.

### Sample preparation for light scattering

Samples for light scattering and small-angle X-ray scattering were prepared in the following manner: A frozen αBC stock solution in sodium phosphate buffer (20 mM sodium phosphate, 0.2 mM EDTA, 0.02% NaN_3_, pH 8.0) at a concentration of 69 μM was thawed and diluted in low-binding Eppendorf tubes to the following concentrations: 10 μM, 22 μM, 34 μM, 46 μM, and 58 μM. The same day approximately 300 μL were transferred to clean 5 mm diameter NMR tubes and sealed with parafilm. The samples were stored at room temperature (approximately 22 °C) for the duration of the experiment.

### Light scattering

Static (SLS) and dynamic (DLS) light scattering experiments were performed on a 3D LS spectrometer (LS Instruments, AG) equipped with a cobolt laser with maximum power of 100 mW and a wavelength of *λ* = 660 nm. The scattered intensity and correlation functions were recorded from 60° to 140° with 5° steps. The measurements were performed at 25 °C in 5 mm diameter glass tubes, emerged in a refractive index-matching liquid (decalin). The sample with the lowest concentration, 10 μM αB-crystallin, displayed a low signal-to-noise ratio resulting in a significant uncertainty in the DLS experiments. Therefore only SLS data for this sample was considered.

The output of the dynamic light scattering (DLS) is an intensity–intensity correlation function *g*^(2)^(*q*, *τ*), where1
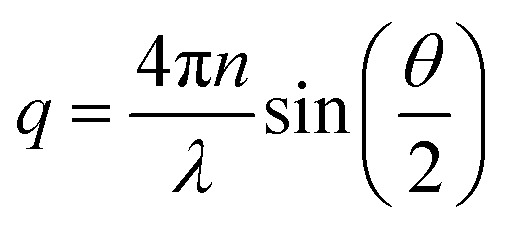
is the scattering vector and *τ* is the lag time. The refractive index of the solution is *n* and *θ* is the scattering angle. From the intensity correlation function one can obtain the diffusion coefficient *D* through the relation2
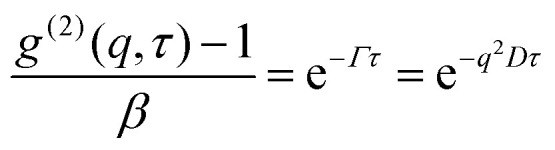
where *β* ≲ 1 is an instrument constant and *Γ* = *q*^2^*D* is the *q*-dependent decay rate. The diffusion coefficient is further related to the hydrodynamic radius, *R*_H_, *via* Stokes–Einstein equation, valid at low concentrations3
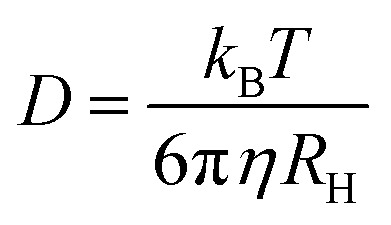


Here, *k*_B_ = 1.38 × 10^−23^ J K^−1^ is the Boltzmann constant, *T* is the temperature and *η* is the solvent viscosity. The diffusion coefficient for each sample was estimated from a linear fit of *Γ*, obtained from a mono-exponential fit, *vs. q*^2^.

Static light scattering (SLS) yields information about the molecular weight of the oligomers. The output of the experiment is the *q*-dependent time-averaged scattering intensity, *I*(*q*). By calibration with toluene, the scattering intensity was transformed to absolute scale, the so-called excess Rayleigh ratio, Δ*R*, by4
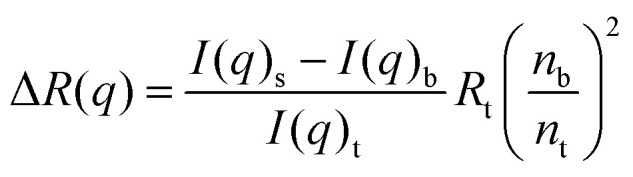
where *I*(*q*)_s_, *I*(*q*)_b_ and *I*(*q*)_t_ are recorded values of scattered intensities from the sample, buffer and toluene, respectively, *R*_t_ is the Rayleigh ratio of toluene, which at 25 °C equals to 1.148 × 10^−5^ cm^−1^. The refractive indices of buffer (water) and toluene are *n*_b_ = 1.33 and *n*_t_ = 1.49, respectively.

### Small-angle X-ray scattering

The samples for small-angle X-ray scattering (SAXS) experiments were taken from the same tubes as the samples for light scattering experiments. Approximately 40 μL of samples was transferred to disposable quartz capillaries on day 7 after thawing and the capillaries were mounted on the SAXS instrument directly after.

SAXS measurements were performed using the laboratory-based Xeuss 3.0 instrument (Xenocs, France) at the center for scattering methods (CSM) in the Faculty of Science, Lund University. The instrument is equipped with a MetalJet X-ray source (Excillum) and the two dimensional scattering patterns were recorded with an Eiger2 R 1M detector (Dectris) and azimuthally integrated using the XSACT software available with the equipment creating one dimensional scattering curve, obtaining the radially averaged intensity, *I*(*q*), where *q* is given by eqn (1), with *n* = 1. The measurements were performed at room temperature at two sample-to-detector distances, 1.8 m and 0.5 m. Absolute scaling of the scattered intensity is provided within the XSACT data reduction from the known scattering volume and the additional recording of the transmitted beam intensity. The data were analyzed using the SasView software (https://www.sasview.org). Pair-distance distribution functions^31^ were calculated from the SAXS data, again using the SasView software.

### Microfluidic diffusional sizing (MDS)

Frozen αBC-NCys-Alexa647 (8 μM) was thawed and diluted to 1 nM, 3 nM, 5 nM, 8 nM, 10 nM, 15 nM, 20 nM, 30 nM, 50 nM, 100 nM, 250 nM, 500 nM, 1 μM, 3 μM, 5 μM, and 8 μM in 20 mM sodium phosphate, 0.2 mM EDTA, 0.02% NaN_3_, pH 8.0 in Eppendorf low-binding tubes. The hydrodynamic radius was calculated using the Stokes–Einstein relation, see eqn (3), by measuring the fluorescence intensities in each chamber post-diffusion (Fluidity One-M, Fluidic Sciences).^32,33^

Measurements were made immediately upon dilution (day 1), after 3 days (day 4), and after a week (day 7). Further information on the time-dependence of the MDS measurements can be found in Section S3.2 in the SI. The samples were stored in the dark at room temperature between the measurements. The measurements were performed by first adding 4 μL of the 20 mM sodium phosphate buffer to each buffer well, waiting for 90 seconds, whereafter 4 μL sample was added to the sample wells. All measurements were done in triplicates. Flow-rate setting 4 (suitable for particles of size 3 nm – 17 nm) and viscosity setting 1 (suitable for low-viscosity liquids in the range 0.82 mPa*s–1.08 mPa*s) were used. The replicates in the concentration range 1–8 μM and the replicates in the concentration range 1–4 nM were pooled together, respectively, to obtain the mean value and standard deviation of *R*_H_ of the plateaus in the MDS data. Data analysis was performed using Graphpad Prism (version 10.3.1 for Windows, GraphPad Software, Boston, Massachusetts USA, https://www.graphpad.com).

The concentrations of the more dilute αBC samples (1 nM–0.5 μM) were internally corrected for the loss of protein through adsorption to various surfaces by using the intensity values from the MDS for the higher αBC concentration samples (1 μM–8 μM). A linear regression line was fitted to averaged intensity values, across all timepoints, for samples in the range 1 μM–8 μM. The regression line was thereafter used to calculate the concentration of the remaining αBC samples, 1 nM–0.5 μM, using the measured intensity values reported by the MDS instrument. This concentration correction is further described in Section S3.1 in the SI.

### Negative stain transmission electron microscopy

Frozen αB-crystallin with a concentration of 55.6 μM was allowed to thaw at room temperature before dilution to 5 μM in 20 mM sodium phosphate buffer (20 mM sodium phosphate, 0.2 mM EDTA, 0.02% NaN_3_, pH 8.0) in a 1.5 mL low-binding Eppendorf tube. Negative stain grids were prepared by applying 4 μL of sample to glow discharged 300 mesh continuous carbon grids for 2 minutes. The samples were blotted, washed with water, and then stained with 2% (w/v) uranyl acetate. Negative stain imaging was performed at the University of Leeds Astbury Centre using a Talos L120C TEM operated at 120 kV.

213 movies were collected from the negatively stained grids at a nominal magnification of 57 000 yielding a pixel size of 2.5 Å for performing 2D classifications. Each movie was collected as MRC fractions. The CTF parameters for each micrograph were estimated using CTFFIND v4.14.^34^ A small set of particles was manually picked using RELION 4^35^ and extracted particles were used to train automated picking using TOPAZ.^36^ A total of 2 591 728 particles were extracted using a box size of 100 pixels for iterations of 2D classifications. The VDAM 2D classification algorithm was used to separate picking artifacts, leaving 239 530 particles for the subsequent round of 2D classification using EM algorithm.

## Results and discussion

**Fig. 2:**
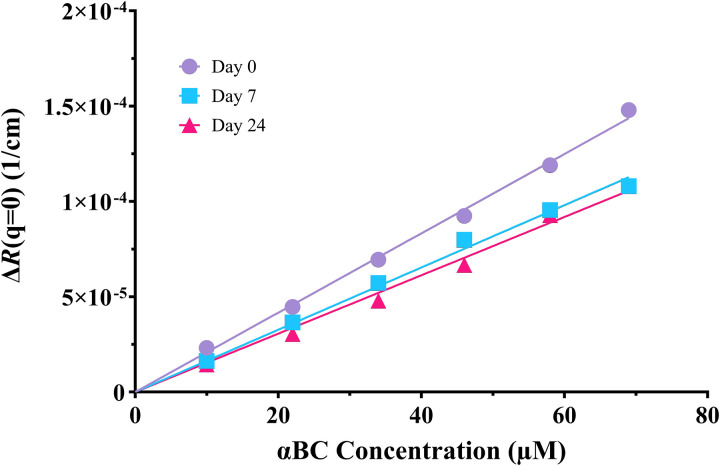
Excess Rayleigh ratio at *q* = 0 *vs.* concentration, measured on day 0 (purple circles), day 7 (blue squares) and day 24 (pink triangles) after sample preparation. The solid lines represent linear fits, yielding an aggregation number *N* = 24 (day 0), *N* = 19 (day 7) and *N* = 17 (day 24). The average final aggregation number is thus *N* = 18.

After thawing and diluting a 69 μM αBC stock solution to 10–58 μM, it was observed through static light scattering that the aggregation number initially decreased with time by approximately 25%, reaching a stable value after about one week. In Fig. 2 the excess Rayleigh ratio, extrapolated to *q* = 0, Δ*R*(0), is presented as a function of the protein concentration, *c*. Here, data from three different incubation times are compared. Measurements labeled as day 0 in Fig. 2 refer to experiments performed on the day of preparation (thawing, dilution). Day 7 and day 24 refer to data recorded after 7 days and 24 days of incubation, respectively. As can be seen, Δ*R*(0) varies linearly with *c*, however the slope is significantly higher day 0 compared to those of days 7 and 24, which are very similar. From this, one can conclude that an equilibration time of approximately one week was sufficient to reach an equilibrium state (Fig. 2). This initial relaxation is likely a consequence of the sample preparation which includes several AMS precipitation steps as well as dissolution and denaturation in guanidine hydrochloride solution just prior to the final SEC step. The short time required for the final SEC step (approximately 1 h), appears not to be sufficient to reach equilibrium of the αBC assemblies. However, as can be seen in Section S2 in the SI, the observation was made that an equilibration time was needed after the final SEC step even for samples that were never frozen after purification. Nevertheless, the assembly state reached after approximately one week is in this study defined as the equilibrium given the conditions, and all measurements reflect this equilibrium state.

For monodisperse assemblies of aggregation number *N*, Δ*R*(0) can be written as5
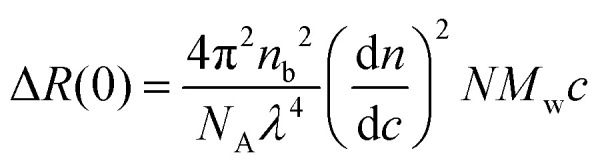
where *N*_A_ = 6.022 × 10^23^ mol^−1^ is Avogadro's number, *n*_b_ is the refractive index of the buffer solvent, *n* the refractive index of the protein solution, 
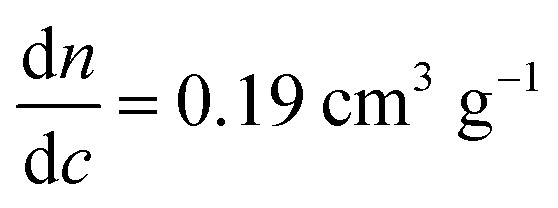
 is the refractive index increment,^37^ and *M*_w_ = 20 × 10^3^ g mol^−1^ is the molar weight of αBC. As can be seen, Δ*R*(0) varies linearly with the protein concentration, which implies that the aggregation number, *N*, is independent of the protein concentration. However, *N* depends on the time after sample preparation. On the day of thawing the stock solution (day 0), *N* = 24 is obtained from the slope in Fig. 2. After one week, *N* had decreased to approximately 18, a value that then remained the same after 24 days. The data thus suggest that *N* = 18 is the equilibrium value that is reached after approximately one week. Therefore, an equilibration time of one week was applied to all other experiments characterizing the self-assembly of αBC.

The aggregation number *N* = 18 found here is smaller than the mean aggregation number found in other studies, although *N* = 18 is still within the range of reported size distributions for αBC. As such, any structural models are for larger assemblies of αBC. Two such models, with *N* = 24, have been obtained from solid state NMR in combination with SAXS and negative stain EM,^16^ as well as from cryo-EM.^19^ However, the equilibration time prior to measurement was not reported in those studies. Interestingly, the difference in aggregation number between these two models and the results found herein is 6 monomers. These 6 monomers could potentially form a hexameric ring, which is one of the suggested building blocks of the αBC oligomer.^18^ It is of note, however, that those structural models were obtained at pH 7.5 while the experiments presented herein were performed at pH 8.0.

**Fig. 3:**
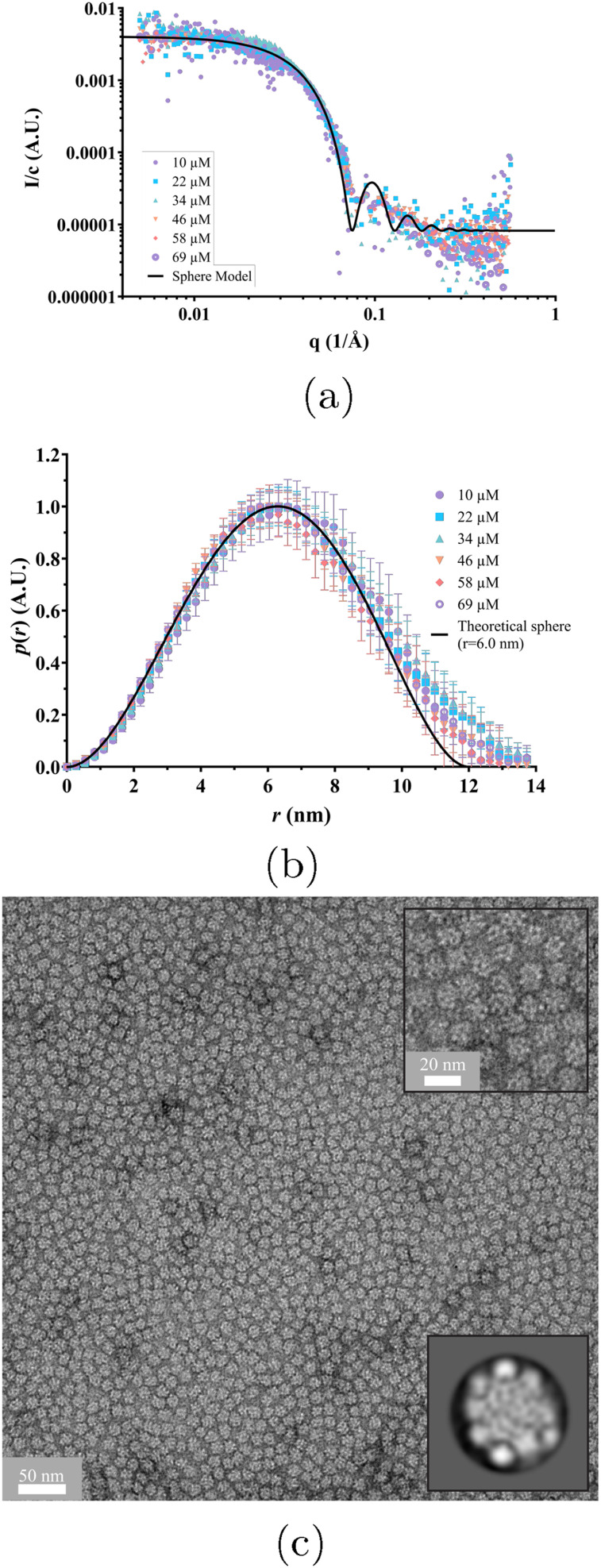
(a) SAXS profiles measured for αBC at several concentrations (see legend) in 20 mM sodium phosphate buffer 7 days after sample preparation. The solid line is a model calculation showing the scattering pattern of homogeneous spheres with a radius of 6 nm. (b) The normalized *p*(*r*) calculated from the SAXS data at the different concentrations (see legend). The solid black line is the theoretical *p*(*r*) curve calculated for a homogeneous sphere of radius 6 nm. (c) Negative stain transmission electron microscopy image of 5 μM αBC in the same buffer. The upper right inset shows a magnified image of αBC. The bottom right inset shows one of the 2D classes generated from the negative stain images. This 2D class is averaged over 2693 particles and has a radius of approximately 7 nm. More information regarding the radius of the particles based on 7 negative stain images can be found in Section S6 in the SI.

In Fig. 3(a) complementary SAXS patterns are presented, obtained from the same samples as were used in the light scattering experiments. The SAXS intensities are all divided by the respective sample concentration, and the fact that the normalized scattering patterns all fall on the same overarching pattern supports the conclusion that the aggregation number, *N*, is independent of the concentration. However, as the scattering pattern also reports on the assembly shape, the data further implies that the assembly shape is independent of the protein concentration.

The SAXS patterns are well described by a simple model of homogeneous spheres, for which the theoretical scattering profile is given by^38^6
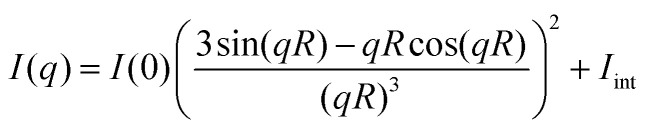
where *I*(0) is the scattered intensity at *q* = 0, and *I*_int_ represents a small and approximately constant intensity resulting from the internal structure of the aggregate and protein molecules. The solid black line in Fig. 3(a) is a calculated scattering pattern using eqn (6) with a radius *R* = 6 nm. As can be seen, there is good agreement between model and data. The oscillations in the model pattern are a consequence of the model's assumption of a sharp discontinuity in the electron density at the sphere-solvent interface, which is not the case for protein assemblies. However, the good agreement between model and data at lower *q*-values imply that the assembly shape of αBC to a good approximation can be described as a sphere. It is also noted that a sphere radius *R* = 6 nm is in good agreement with what has been reported in literature using SANS and SAXS.^39,40^

From comparing the volume of a sphere of radius 6 nm with the total volume of *N* = 18 protein molecules, the volume fraction *f* of proteins in the aggregates can be roughly estimated. Assuming a protein mass density of 1.4 g mL^−1^,^41^*f* is estimated to 0.5, a reasonable value. Notably, despite the estimation that αBC assemblies contain about 50% water, modeling them as homogeneous spheres remains valid, as SAXS profiles show no evidence of internal assembly structure. However, it is worth noting that should there be a small spherical empty space at the center of the oligomer it would not be detectable through SAXS experiments. As such, the possibility of a small empty space in the oligomer center cannot be dismissed. Indeed, a small central cavity has been suggested.^16,19,25,26^

Another convenient way to evaluate aggregate size and shape is to transform the experimental scattering function *I*(*q*) into real space, to obtain the pair-distance distribution function *p*(*r*).^31^ In Fig. 3(b)*p*(*r*), normalized to an amplitude equal to unity, obtained for the different protein concentrations is plotted. As can be seen, *p*(*r*) for the different concentrations are essentially identical. The bell shaped curves imply that the particle shapes are approximately spherical^31^ and the maximum pair-distance is *D*_max_ ≈ 13 nm. For a homogeneous sphere, *p*(*r*) has an analytical expression^31^7
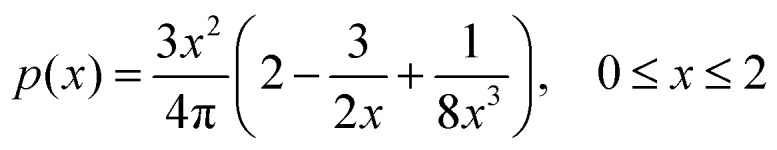
with *x* = *r/R*. As a solid line in Fig. 3(b) a theoretical *p*(*r*) for homogeneous sphere with *R* = 6 nm is plotted. As can be seen, this model describes the data well with only a minor discrepancy for *r* near *D*_max_. This is not surprising, as the aggregates are made up of self-assembled protein molecules. The conclusion, however, is that the aggregates to a good approximation can be described as homogeneous spheres with a radius of 6 nm.

Negative stain TEM was performed with a 5 μM sample, and a representative image is shown in Fig. 3(c). The image shows a large number of αBC oligomers as well as two insets: a higher magnification image of the sample and a generated 2D class. The TEM image confirms the conclusions from SAXS that the aggregates are essentially spherical, with a radius of approximately 7 nm. However, while SAXS data does not indicate any internal structure, the 2D class obtained from the negative stain TEM and presented in Fig. 3(c) shows what resembles a corona at the edges of the assemblies. This would suggest that αBC oligomers are composed of smaller beads. Nevertheless, no further conclusions can be drawn regarding the 3D structure of αBC oligomers. This is due to the fact that the stain may cover the grid with the sample unevenly, leading to artifacts in the 2D class averaging. Additionally, the αBC oligomers may be affected by the harsh conditions that negative staining involves, leading to possible deformations of the shape, in line with what was previously noted by Jehle *et al.*^16^ Despite this, the radius of the 2D class is approximately 7 nm which is comparable to the results of the other methods in this paper. Therefore, the image in Fig. 3(c) can be said to support the proposed idea that αBC oligomers are spherical with a radius of approximately 6–7 nm.

**Fig. 4:**
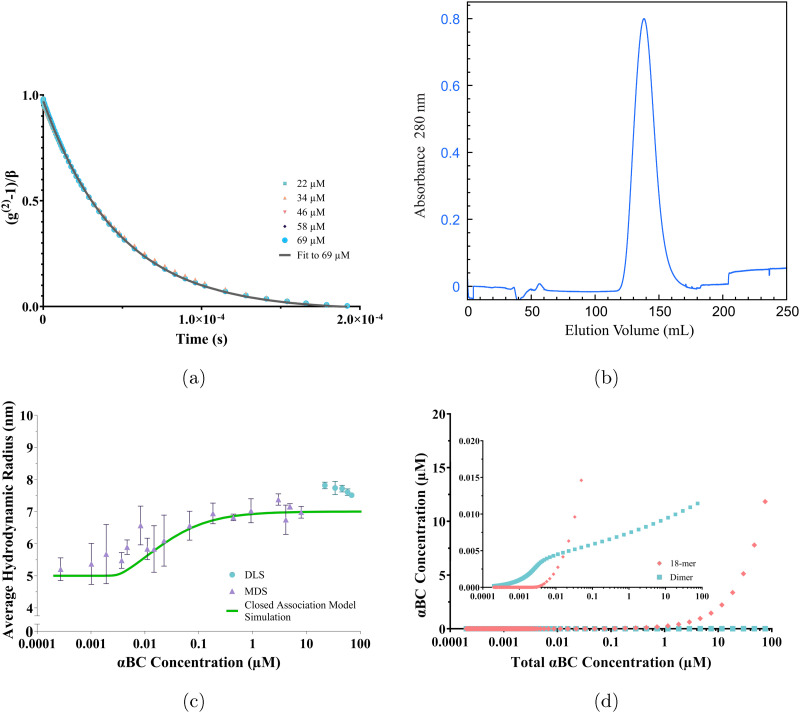
(a) Intensity–intensity correlation functions for different αBC concentrations (see legend inset) measured at 90° on day 7 after sample preparation and a mono-exponential fit (black solid line). The fit yields *R*_*H*_ = 7.4 nm. See Section S4 in the SI for a graph of the same data plotted on a logarithmic *x*-axis. (b) Chromatogram (absorbance at 280 nm) of the second size exclusion in the purification process of αB-crystallin, displaying a narrow peak. (c) *R*_*H*_*vs.* concentration obtained by MDS (purple triangles) and DLS (turquoise circles). The closed association model simulation is shown as a solid green line. (d) Simulations based on the closed association model of the concentration of dimeric αBC (turquoise squares) and the concentration of 18-meric (9 dimeric subunits) αBC (peach rhombi) as a function total αBC concentration. The inset shows a magnification of the concentration behavior at the lower concentrations of αBC. Note the shift in behavior of the dimeric and 18-meric simulation data at approximately 4 nM.

DLS experiments were also performed on the same samples as SLS and SAXS. The normalized intensity correlation functions are presented in Fig. 4(a) (here the data from the 10 μM sample was omitted due to the relatively poor signal-to-noise ratio). As can be seen, the correlation functions from the samples at five different concentrations are essentially identical, again supporting the conclusion that the assembly size is independent of the concentration. The correlation functions furthermore show a single exponential decay. The best fit with a single exponential decay to the 69 μM data is shown as solid line, from which a hydrodynamic radius of 7.4 nm is obtained. This value is in good agreement with previous studies using DLS.^25,26,40^ The single exponential decay implies that the αBC oligomers are essentially monodisperse. A narrow size distribution is also supported by the observation of a narrow elution peak in the SEC chromatogram, see Fig. 4(b).

DLS experiments are limited to relatively high protein concentrations. To study the self-assembly also at sub-micromolar concentrations, diffusion experiments using microfluidic diffusional sizing (MDS) were performed. Using MDS, a wide concentration range was covered, from 1 nM to 8 μM. The data are presented in Fig. 4(c) where the average hydrodynamic radius, *R*_*H*_, is plotted as a function of the protein concentration. At higher concentrations of the MDS data, 1–8 μM, *R*_*H*_ = 7.0 ± 0.3 nm, independent of the concentration, in agreement with the DLS data. Below about 100 nM, *R*_*H*_ decreases gradually with decreasing concentration, and levels off at *R*_*H*_ = 5.4 ± 0.5 nm below approximately 4 nM. Thus, the concentration ≈4 nM marks the onset of αB-crystallin self-assembly into larger oligomers.

The observation of essentially monodisperse oligomers of αBC, with *N* ≈ 18 independent of the protein concentration, implies a very strong preference for this particular aggregation number. To a good approximation the self-assembly can therefore be described as following the closed association model, characterized by a single association constant 
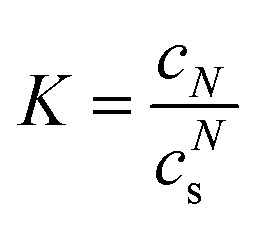
, or dissociation constant 
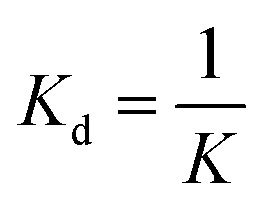
.^42^ Here, *c*_s_ is the subunit concentration and *c*_*N*_ is the concentration of the *N*-mer, *N* now being the number of subunits in the oligomer. For larger *N*-values this model predicts an onset of aggregation at a critical concentration 
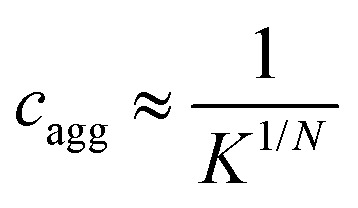
. For *c* < *c*_agg_ essentially only subunits are present. For *c* > *c*_agg_, *c*_s_ varies (increases) only weakly with increasing *c*. At higher concentrations *c* ≫ *c*_agg_, assemblies dominate and subunits can be neglected.

The value of *R*_H_ ≈ 5 nm at lower concentrations, below *c*_agg_, is larger than what is expected for an αB-crystallin monomer, suggesting that the subunit also is in an oligomeric state. In line with previous work,^15,17,18,20–23^ it is here assumed that the subunit is a dimer. With only dimers and the larger assemblies, built of *N* = 18/2 = 9 subunits, present, the total (protein) concentration is given by *c* = 2(*c*_s_ + *Nc*_*N*_).

In Section S5 of the SI an approximate expression for 〈*R*_*H*_〉 in a polydisperse system is derived for the MDS experiment. For a bimodal size distribution, subunits and the larger assemblies, the expression is8
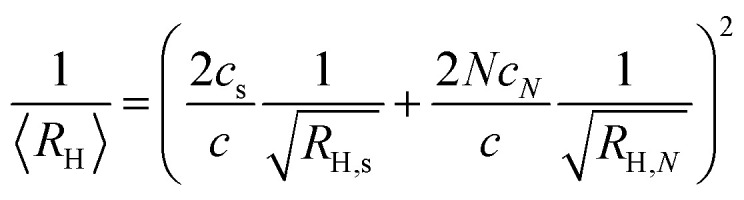


Here, *R*_*H*,*s*_ and *R*_*H*,*N*_ are the hydrodynamic radii of the subunit and larger *N*-mer, respectively. Within the closed association model, the variation of *c*_s_ and *c*_*N*_ as a function of the total protein concentration *c* can be calculated for given value of *N* and *K*. Having the values of *c*_s_ and *c*_*N*_, 〈*R*_*H*_〉 in the MDS experiment can be calculated from eqn (8). With *N* = 9 and assuming *R*_*H,s*_ = 5 nm and *R*_*H*,*N*_ = 7 nm, *K* is adjusted to obtain a curve, *R*_*H*_*versus c*, that roughly describes the experimental data. Based on this, a calculation using *K* = 10^−4^ nM^−8^ is shown as a solid green line in Fig. 4(c). The calculation should be seen as an estimate only of the association constant. The small difference in *R*_*H*_ between the larger oligomers and the subunits makes experimental uncertainties relatively large. In any case, the parameter values used in the calculations have been adjusted to agree with the experimental data, and suggest that there is an onset of aggregation at *c*_*agg*_ ≈ 4 nM. Below this concentration 2*c*_*s*_ essentially equals *c*. Above this concentration the subunit concentration varies only weakly with increasing *c*. Increasing *c* > *c*_*agg*_ results mainly in an increase of *c*_*N*_. This can be seen in Fig. 4(d), where the protein concentrations in subunits (2*c*_*s*_) and larger oligomers (18*c*_*N*_), respectively, are plotted as a function of the total protein concentration.

The self-assembly of αB-crystallin studied in this work is an example of protein aggregation and is thus related to amyloid formation and the aggregation of misfolded or denatured proteins. However, there is an important fundamental difference. αB-crystallin self-assembles above a semi-critical concentration, *c*_*agg*_ ≈ 4 nM, into finite sized aggregates, here found to consist of approximately 18 αB-crystallin monomers. This is to be compared with the practically infinite aggregates formed by amyloid proteins. For amyloid-β40 and 42 (Aβ40 and Aβ42) at 37 °C and pH 7.4 or 8.0, respectively, the corresponding critical aggregation concentrations, viewed as monomer solubility, were found to be approximately 400 nM^43,44^ and 30 nM,^45^ respectively. The fact that the chaperone aggregates are finite is interesting. It allows the protein to have a partial hydrophobic character in the form of hydrophobic patches, important for its action, and be present at higher concentrations without precipitating out of solution with possibly toxic larger aggregates.

## Conclusions

In this work, the molecular chaperone protein αB-crystallin has been characterized at pH 8.0 at concentrations spanning almost 5 orders of magnitude (1 nM to 69 μM). Through small-angle X-ray scattering and negative stain transmission electron microscopy at concentrations ≥5 μM, αB-crystallin oligomers are shown to assume a homogeneous quasi-spherical 3D structure where the protein molecules are distributed homogeneously throughout the oligomers. These quasi-spherical αB-crystallin oligomers were found to have an effective radius of 7 nm which is in good agreement with the diffusion experiments, microfluidic diffusional sizing and dynamic light scattering, which collectively suggest a hydrodynamic radius of approximately 7 nm. Further conclusions about αB-crystallin's three-dimensional structure cannot be drawn and thus require other methods.

Regarding the size distribution of αB-crystallin oligomers, small-angle X-ray scattering, negative stain transmission electron microscopy images, microfluidic diffusional sizing, size exclusion chromatography, as well as static and dynamic light scattering all indicate that αB-crystallin has a monodisperse-like behavior. It is found that αB-crystallin forms large oligomers of approximately 18 monomers, in agreement with the range reported by previous studies and the size is remarkably independent of the protein concentration. Based on the data presented in this study, it is here proposed that the self-assembly of αB-crystallin can, to a good approximation, be described using the closed association model, with a critical aggregation concentration of approximately 4 nM.

At low concentrations, the microfluidic diffusional sizing data indicate a dissociation of the oligomers into subunits having a hydrodynamic radius of 5.4 ± 0.5 nm. This is larger than what is expected for a partially folded monomer.^46^ In line with previous work,^15,17,18,20–23^ it is here assumed that the subunits are dimers.

From analyzing the low concentration microfluidic diffusional sizing data and assuming the closed association model to apply for the oligomers of 9 dimer subunits, the association constant is estimated to be *K* ≈ 10^−4^ nM^−9^, and an onset of aggregation to be approximately 4 nM.

Additionally, it is of note that the samples in this study required an equilibration time, where approximately one week after thawing was sufficient in order to reach an equilibrium size distribution. This equilibration time likely depends on the details of the sample preparation protocol, and the importance of monitoring samples over time is here stressed to confirm that they are in an equilibrium state.

Finally, these findings are an important step to understanding one of the body's own protection systems – the chaperone system – toward protein misfolding diseases such as Alzheimer's. The same underlying interactions may drive client-chaperones co-assembly and chaperone self-association. While the aggregation of client proteins is infinite and toxic, the self-association of chaperones is finite and non-toxic. First understanding the chaperone self-association may facilitate insights into the more complex client-chaperone association to stimulate new therapeutic strategies for treating neurodegenerative diseases, for example deriving chaperone mimics.

## Author contributions

All authors contributed equally.

## Conflicts of interest

The authors have no conflicts of interest to declare.

## Supplementary Material

SM-021-D5SM00684H-s001

## Data Availability

Supplementary information is available. See DOI: https://doi.org/10.1039/d5sm00684h All data presented in this paper have been deposited onto Github for public access and can be found by following this URL: https://github.com/saralinse/Published_Data/tree/Soft_Matter_2025_aBC_self-assembly.
