# Supplementary material for: On the self-assembly of αB-crystallin
Source: Soft Matter. 2025 Aug 25;21(37):7308–17. doi: 10.1039/d5sm00684h (PMC12403594; doi:10.1039/d5sm00684h)
Supplement: SM-021-D5SM00684H-s001 [file SM-021-D5SM00684H-s001.pdf]

Supplementary Information (SI) for Soft Matter.
This journal is © The Royal Society of Chemistry 2025

Supporting Information: "On the Self-Assembly of αB-Crystallin"

Ewelina Lindbladh[a,*,†], Marija Dubackic[b,*,†,‡], Dev Thacker[a,c], Sara Linse[a], Ulf Olsson[b]

[a]Biochemistry and Structural Biology, Chemical Center, Lund University, Lund, Sweden
[b]Physical Chemistry, Chemical Center, Lund University, Lund, Sweden
[c]Astbury Centre for Structural Molecular Biology, University of Leeds, Leeds, United Kingdom

† These authors contributed equally and share first authorship.
*Corresponding authors: ewelina.lindbladh@biochemistry.lu.se
majuskad@hotmail.com
‡ *Current address: RISE PFI AS, Høgskoleringen 6B, Trondheim 7491, Norway*

S1. αB-Crystallin Protein Expression and Purification

Here follows further details for the expression of αB-crystallin (αBC), which expands on the information given in the section *Protein Expression, Purification, and Sample Preparation* in the main text. In Figure 1 below the different domains, the amino acid sequence, as well as the DNA sequence annotated with restriction enzyme recognition sites and stop codon positions, used for αBC are illustrated.

(a)

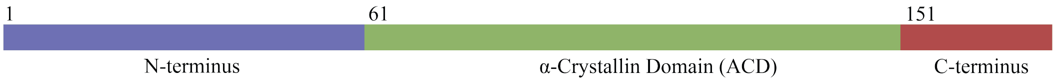

(b)

1 MDIAIHHPWIRRPFFPFHSPSRLFDQFFGEHLLESDLFPTSTSLSPFYLR 50

51 PPSFLRAPSW 61 FDTGLSEMRLEKDRFSVNLDVKHFSPEELKVKVLGDVIEV 100

101 HGKHEERQDEHGFISREFHRKYRIPADVDPLTITSSLSSDGVLTVNGPRK 150

151 QVSGPERTIPITREEKPAVTAAPKK 175

(c)

5'-1 CATATGGATATCGCTATCCACCACCCGTGGATTCGTCGTCCGTTCTTCCCGTTCCACTCTCCGTCTCGTCTGTTCGACCAGT 82

83 TCTTCGGTGAACACCTGCTGGAATCTGACCTGTTCCCGACCTCTACCTCTCTGTCTCCGTTCTACCTGCGTCCGCCGTCTTTCCT 167

168 GCGTGCTCCGTCTTGGTTCGACACCGGTCTGTCTGAAATGCGTCTGGAAAAAGACCGTTTCTCTGTTAACCTGGACGTTAAACAC 252

253 TTCTCTCCGGAAGAACTGAAAGTTAAAGTTCTGGGTGACGTTATCGAAGTTCACGGTAAACACGAAGAACGTCAGGACGAACACG 337

338 GTTTCATCTCTCGTGAATTCCACCGTAAATACCGTATCCCGGCTGACGTTGACCCGCTGACCATCACCTCTTCTCTGTCTTCTGA 422

423 CGGTGTTCTGACCGTTAACGGTCCGCGTAAACAGGTTTCTGGTCCGGAACGTACCATCCCGATCACCCGTGAAGAAAAACCGGCT 507

508 GTTACCGCTGCTCCGAAGAAATAGTAAGGATCC 540 -3'

Figure 1: (a) The domain distribution of αBC. Blue corresponds to the N-terminus, green to the α-crystallin domain, and red to the C-terminus. (b) The amino acid sequence of αBC used in this study. The color corresponds to the different domains of the protein as shown in (a). (c) The DNA sequence for αBC that was used for expression. The recognition sites for *NdeI* and *BamHI*, the restriction enzymes used for ligation of the gene into the Pet3a vector, are underlined at positions 1-6 and 535-540, respectively. The two stop codons used are highlighted in red. This gene sequence with the GAT codon for D2 gives very high expression of αBC, whereas a gene with GAC codon for D2 gives virtually no expression, likely due to unfavorable mRNA secondary structure [1].

As can be seen in Figure 2, αBC elutes in a relatively narrow peak in anion exchange chromatography (IEX) and in both rounds of size exclusion chromatography (SEC1 and SEC2). A low denaturant concentration (1.5 M GuHCl) in the first SEC is used to dissociate oligomers and αBC elutes late, followed by native SEC in which αBC elutes earlier as oligomer, thus leading to higher

purity compared to using a single condition. Only the fractions between the black lines in the chromatograms were used for further purification and analysis. The narrow chromatogram profiles indicate that αBC adopts a narrow size distribution, in line with the results obtained by the other methods presented in this paper. Additionally, the corresponding gels of fractions collected at the chromatogram peaks indicate a pure αBC preparation. In the IEX and SEC1 (Figure 2(a)-(b)) faint bands corresponding to a size to that of an αBC dimer can be discerned, however, these bands are not present in the gel of the second size exclusion step (Figure 2(c)).

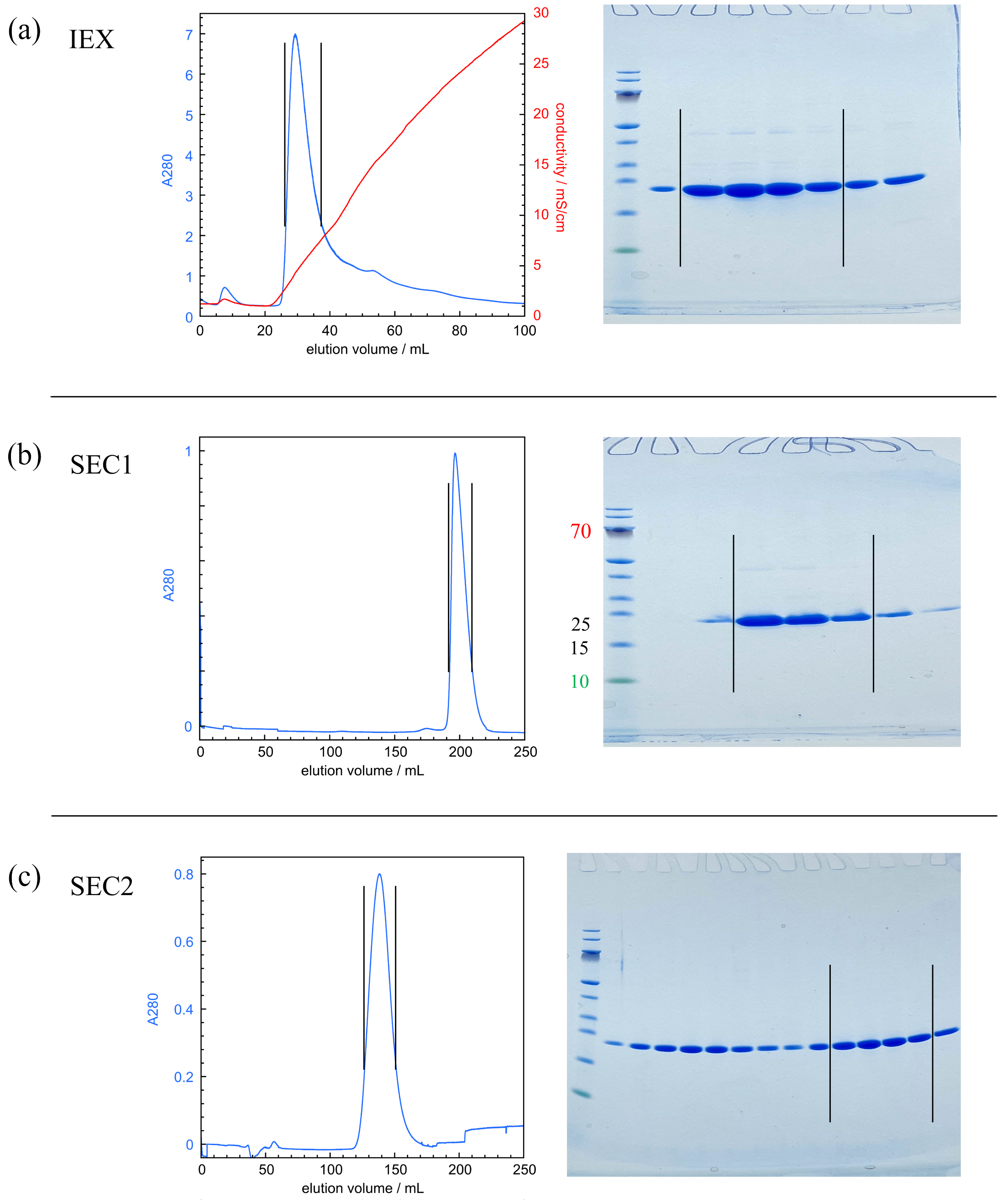

Figure 2: Chromatograms and SDS-PAGE gels from different steps of the purification process. Across panels (a)-(c) the blue traces correspond to the absorbance at 280 nm. (a) Anion exchange chromatogram and SDS-PAGE gel. The red trace shows the conductance. The vertical black lines in the chromatogram and on the gel indicate which fractions were used for further purification. (b) The chromatogram and SDS-PAGE gel of the first size exclusion chromatography on a Superdex 200 (26/600) column. The buffer used in this step was 20 mM sodium phosphate, 0.2 mM EDTA and 1.6 M GuHCl at pH 8.0. The vertical black lines in the chromatogram and gel indicate which fractions were used for further purification. (c) The chromatogram and SDS-PAGE gel of the second size exclusion chromatography on a Superdex 200 (26/600) column. The buffer used was 20 mM sodium phosphate and 0.2 mM EDTA at pH 8.0. The vertical black lines in the chromatogram and on the gel indicate which fractions were saved for further studies. The gel in panel (c) includes fractions from two replicates of SEC 2 and the fractions from the second replicate were used in this study.

S2. On the Equilibration of the Assembly Size

The measurements using microfluidic diffusional sizing and dynamic light scattering showed relaxation of oligomer size over time after thawing of purified αB-crystallin, which prompted a small investigation into the reasons for this effect. To exclude that it was an artifact of freezing the purified αBC, one batch of αBC was purified according to the same protocol as previously described. However, αBC was collected immediately upon eluting from the second size exclusion column and brought to the dynamic light scattering instrument. Equivalent measurements were made for this sample of non-frozen αBC as the ones performed for the frozen and thawed αBC, which are presented in Figure 3. The data show that the same behavior is displayed by non-frozen αBC as thawed αBC, and thus it is unlikely that the relaxation behavior is an artifact of the freezing and thawing of αBC samples.

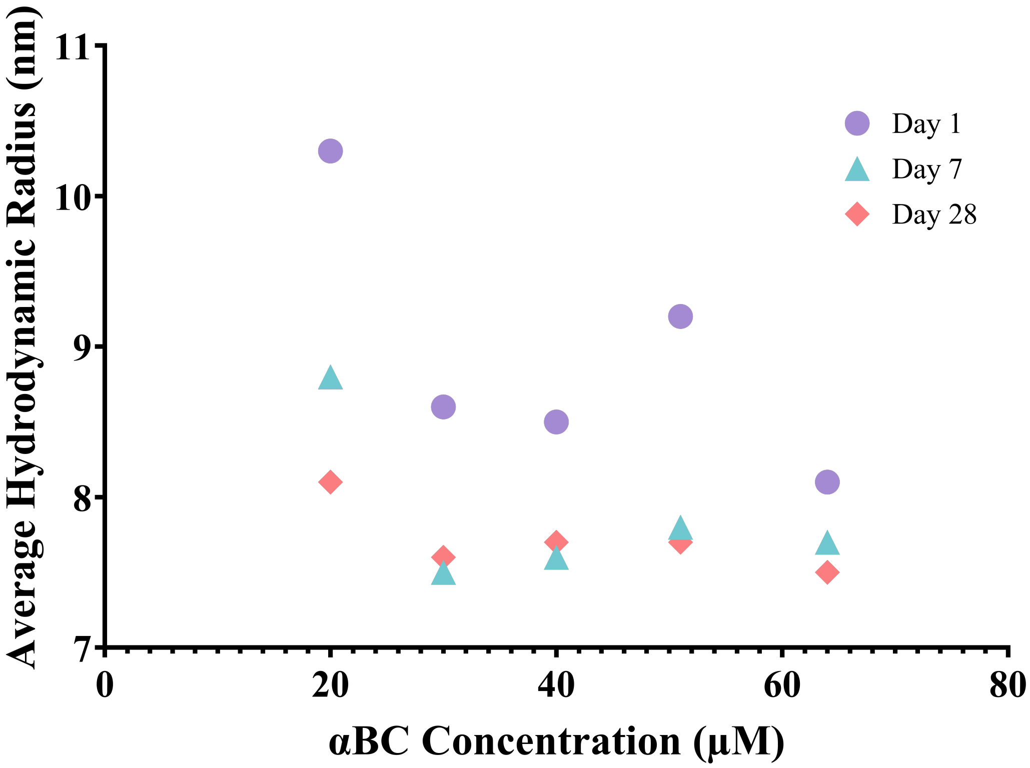

Figure 3: Hydrodynamic radius as a function of total αB-crystallin concentration over time. See legend inset for how many days after collection from the SEC each measurement was performed. Note that the biggest shift in behavior occurs from Day 1 to Day 7, and the radius does not change much between Day 7 and Day 24.

However, it should be noted that despite the fact that the relaxation time does not appear to be dependent on freezing and thawing the sample, it cannot be excluded that other steps in the expression and purification of αBC is not a factor. On the other hand, relaxation of the αBC assembly size may also be an inherent property of the protein.

S3. Microfluidic Diffusional Sizing

S3.1. Concentration Correction

The microfluidic diffusional sizing (MDS) instrument reports on both the measured intensity as well as the average hydrodynamic radius (R_H) for the sample. To assess the linear range of of the instrument for αBC samples the correlation between concentration and measured intensity was studied. Linear regression was performed for each timepoint dataset using GraphPad Prism (version 10.3.1 for Windows, GraphPad Software, Boston, Massachusetts USA, www.graphpad.com) and is presented in Figure 4. The linear regression analysis yielded high R^2 values for all timepoint datasets, supporting the validity of the measured R_H values at these concentrations.

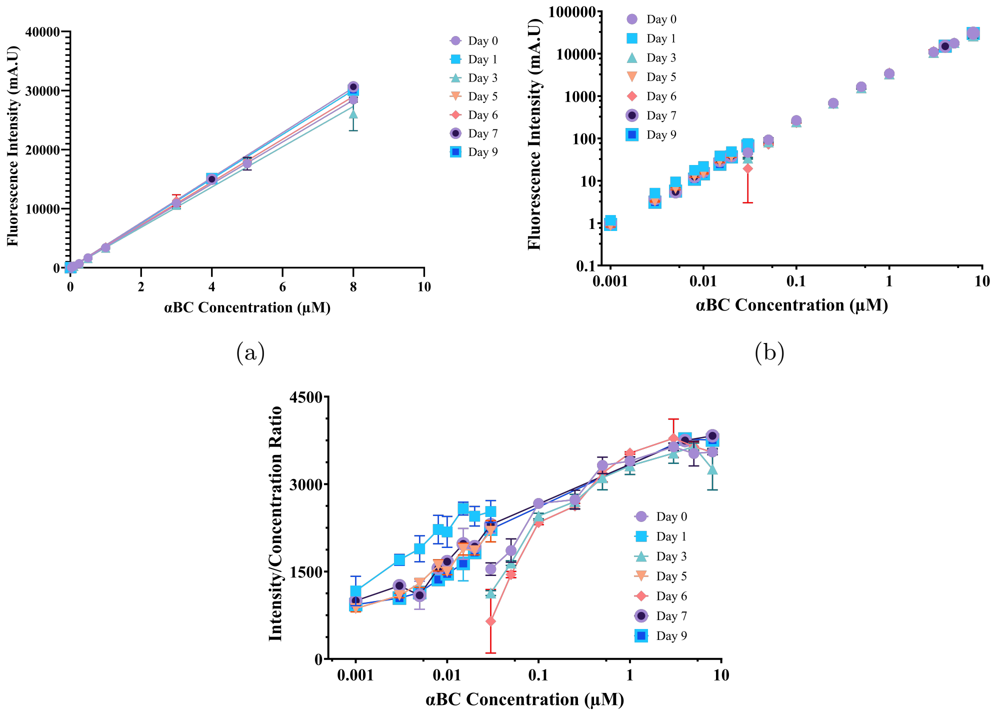

(a) (b)

(c)

Figure 4: The correlation between the concentration of αBC and the intensity readout in the microfluidic diffusional sizing instrument. See legend insets for which data corresponds to the different timepoints in panel (a), (b), and (c), respectively. (a) The intensity as a function of αBC concentration at different timepoints after dilution. The R^2 values of the trendlines are: Day 0, 0.9982 ; Day 1, 0.9837 ; Day 3, 0.9904 ; Day 5, 0.9631 ; Day 6, 0.9974 ; Day 7, 0.9998 ; Day 9, 0.9999. (b) The same data as in (a) but plotted on a logarithmic axes. (c) The ratio of the measured intensity over the sample αBC concentration.

However, it was noted that the data presented in Figure 4 does not follow a 1:1 relationship between fluorescence intensity and αBC concentration despite high R^2 values, which prompted an analysis of the ratio between fluorescence intensity and concentration. This is presented in Figure 4(c). In a model system the ratio between measured intensity and concentration should be a constant value, but as is seen in Figure 4(c) the ratio appears to systematically decrease with decreasing αBC concentration. This points to a loss of αBC in either sample preparation or in the MDS chip.

As a consequence, the concentrations of the samples that were measured in the MDS is lower than anticipated. To assess the extent of lost αBC, the concentrations of the lower αBC concentrations were internally corrected using the higher αBC concentrations. As is described under *Microfluidic Diffusional Sizing (MDS)* in *Methods*, the αBC concentrations 1 µM - 8 µM served as the internal standards. A linear regression line was fitted to the average value of each concentration across all timepoints and was used to calculate the true concentrations of the samples with nominal concentrations of 1 nM - 0.5 µM. For a visual representation of the correction, see Figure 5.

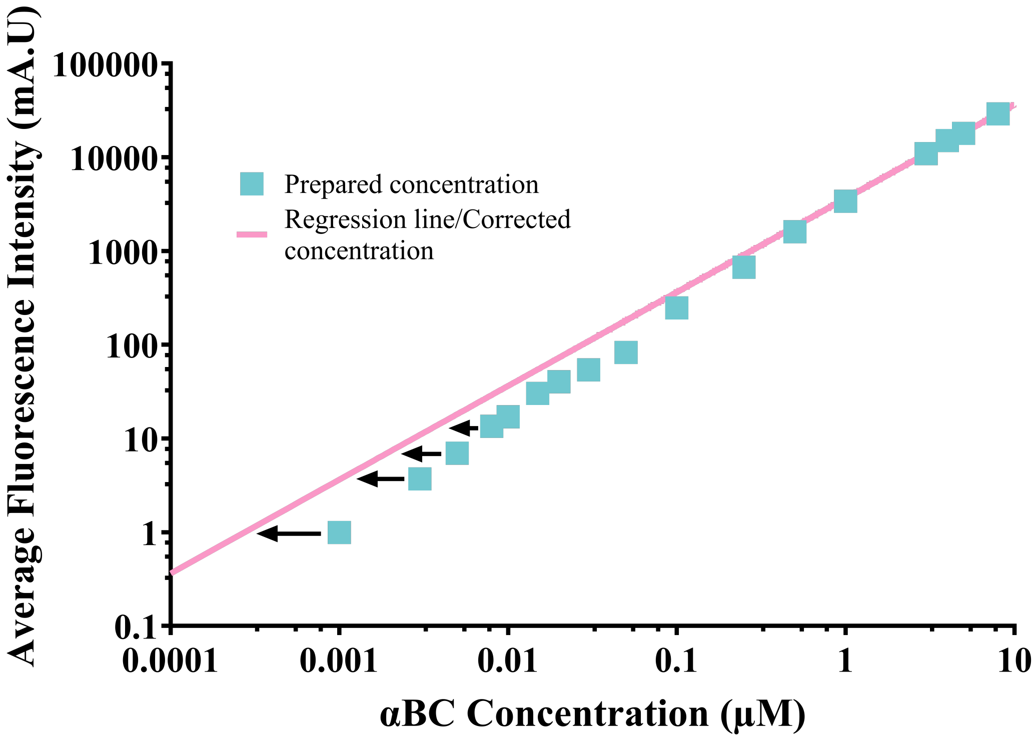

Figure 5: Visualization of the method and effect of the concentration correction process. Four averaged data points from the MDS data corresponding to 1 µM, 3 µM, 5 µM, and 8 µM were used for linear regression with the constraint that it had to pass through (0,0). The regression line, y=3636.796957x, is colored in light pink. The regression line was then used to go from prepared concentration (blue squares) to the corrected concentration using the average fluorescence intensities as indicated by the black arrows.

S3.2. Time Dependence

Prior to any measurements were performed the evolution of the average hydrodynamic radius over time was assessed. The average R_H was followed over 9 days, and as can be seen in Figure 6, lower concentrations appear to measure around 7.5 nm immediately after thawing and diluting. The size then decreases over time, and 9 days after thawing and diluting it measures approximately 5 nm in radius. This trend is mostly seen for αBC samples with low concentrations. For instance, the 0.001 µM sample displays this clear decrease, whereas the 8 µM sample shows quite consistent values at all timepoints. In Figure 6(a) it can be seen that the average R_H appears to reach a plateau value after approximately 5-7 days. This observation served as the basis to perform all subsequent measurements after at least 5 days of incubation in room temperature. However, the relaxation to an equilibrium size appears to be rather slow. Therefore, this incubation time should be treated with caution, as the true equilibrium size might not yet have been reached.

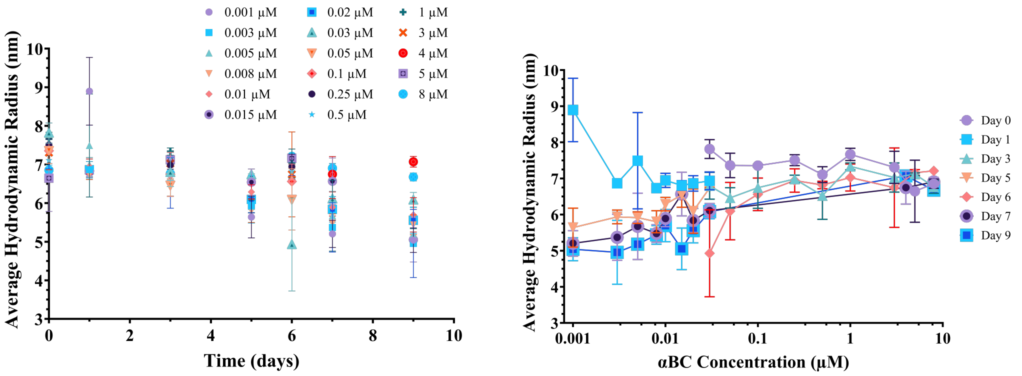

(a) (b)

Figure 6: The dependency of the average hydrodynamic radius on time and concentration. See legend insets for which data corresponds to which concentration or timepoint in (a) and (b), respectively. (a) The different sample concentrations average R_H values as a function of time. (b) How the average R_H changes depending on the sample concentration, followed at different timepoints.

S4. Dynamic Light Scattering Correlation Graph

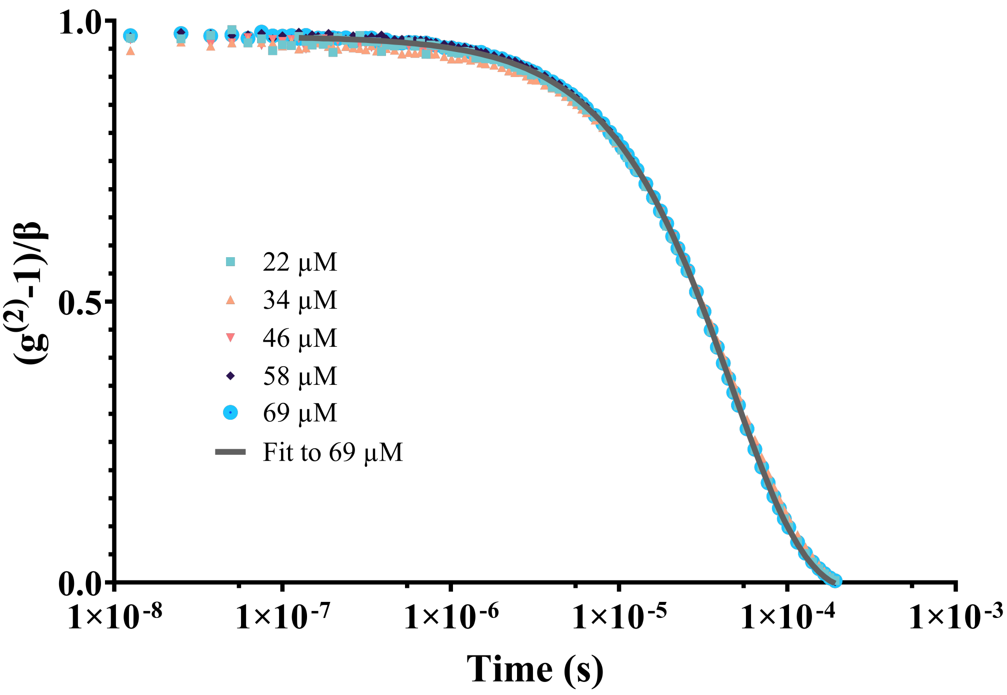

Figure 7: Intensity-intensity correlation functions for different αBC concentrations (see legend inset) plotted on a logarithmic x-axis to complement Figure 4(a) in the main paper. This data was measured at 90 ° on Day 7 after sample preparation and a mono-exponential fit (black solid line). The fit yields $R_H = 7.4$ nm.

S5. A Note on Average Hydrodynamic Radius Calculations in Microfluidic Diffusional Sizing

The microfluidic diffusional sizing (MDS) technique is based on the laminar flow between two exposed volumes: one containing the analyte and the other being the buffer that the analyte is dissolved in. Due to the flows of these volumes being laminar in nature, when exposed to each other only diffusion will drive the transport of analyte into the buffer flow. For a schematic of this process, see Figure 8(a). At the end of the microfluidic chip there are two chambers. The intensity of the analyte is measured in both of these chambers at the end of the experiment.

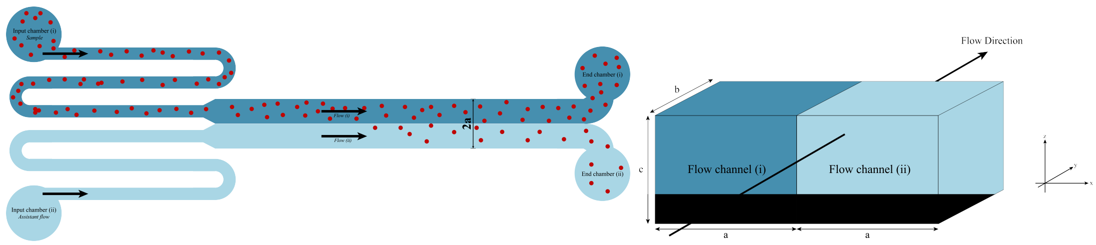

Figure 8: Schematic of the microfluidic diffusional sizing principle as well as a defined volume element within the microfluidic chip. (a) Schematic of the flow in the microfluidic chip. Sample ((i)) and an assistant liquid ((ii)) is added to their respective chambers, and these flows are later exposed to each other. Because the flows are laminar, only diffusion drives the transfer of particles (red spheres) from one flow to the other. The intensity of the particles are measured in end chambers (i) and (ii), upon which the average hydrodynamic radius is calculated. (b) A volume element of the sample and assistant flows when they are exposed to each other in (a). The channels (i) and (ii) are exposed to each other in the yz-plane and the flow of samples occur along the y-axis. Diffusion therefore occurs along the x-axis. Both channels are defined as having a volume of $V_1 = V_2 = abc$, and the contact area between the two flows are defined as $A = bc$.

At a given point during the experiment, a volume element can be defined as in Figure 8(b). Channel (i) refers to the flow containing the analyte whereas channel (ii) refers to the flow only containing buffer. The direction of the flow in the chip is indicated by the black arrow. The flow can be assumed to follow Fick's first law in one dimension (x), see Equation 1. Fick's first law describes the net flux J (unit: $\frac{1}{area*time}$), where D is the diffusion coefficient and the sign reflects the direction of the flux.

$$J = -D\frac{dc}{dx} \tag{1}$$

Fick's second law, describing the time dependence of the local concentration, can also be assumed to apply to the system. See Equation 2. However, at short time intervals the steady-state approximation ($\frac{\partial c}{\partial t} = 0$) can be applied. The second derivative of the steady-state approximation allows for deducing the concentration gradient. The second derivative becomes 0, indicating that the concentration gradient in the x-dimension is linear. Hence, $c(x) = c(0) + kx$.

$$\frac{\partial c}{\partial t} = D\frac{\partial^2 c}{\partial x^2} \tag{2}$$

At t=0 ther are only particles in channel (i), with a concentration of $c_\alpha = c_0 = \frac{n}{V}$, where n is the total number of particles. With time, particles diffuse over to channel (ii). Denoting the number of particles in the two channels $n_{(i)}$ and $n_{(ii)}$, respectively, with $n_{(i)} + n_{(ii)} = n$, the flux can be defined as in Equation 3.

$$J = \frac{1}{A}\frac{dn_2}{dt} = -D\frac{dc}{dx} \tag{3}$$

Due to the linearity of the concentration gradient, the slope can be defined as $\frac{dc}{dx} = -\frac{c_0}{2d}$ as $x = -d$ to $x = d$ over the two laminar flows depicted in Figure 8. Together with the expression of the concentration dependence stated above, the concentration profile can be defined as the first part of Equation 4 below. The number of particles existing in channel (ii) then becomes the second part of Equation 4.

$$c(x) = c_0 - \frac{c_0}{2d}x \Rightarrow n_{(ii)} = \frac{Adc_0}{4} \tag{4}$$

Combining the expression for the slope ($\frac{dc}{dx} = -\frac{c_0}{2d}$) of the concentration profile and Equations 3 and 4, an expression for the change of particles in channel (ii) over time is obtained. By applying a boundary condition, $n_{(ii)} = 0$, the differential equation in Equation 5 can be solved.

$$\frac{dn_{(ii)}}{dt} = \frac{DA^2c_0^2}{8n_{(ii)}} \Rightarrow \Bigg/ \frac{dc}{dx} = -\frac{c_0}{2d} \Bigg/ \Rightarrow n_{(ii)}(t) = \frac{Ac_0}{2}\sqrt{Dt} \tag{5}$$

Having established the change of particles in channel (ii) over time, the diffusion coefficient can be evaluated from the ratio of the particle concentrations in both channels ($c_{(i)}$ and $c_{(ii)}$). This derivation is shown in Equation 6 below where a is defined as $a = \frac{V}{A}$.

$$n_{(ii)}(t) = \frac{Ac_0}{2}\sqrt{Dt} \Rightarrow \Bigg/ \frac{c_{(i)}}{c_{(ii)}} = \frac{n_{(i)}}{n_{(ii)}} = \frac{n - n_{(ii)}}{n_{(ii)}} = \frac{c_0V}{n_{(ii)}} - 1 \Bigg/ \Rightarrow D = \frac{1}{t}\left(\frac{2a}{1 + \frac{c_{(i)}}{c_{(ii)}}}\right)^2 \tag{6}$$

For illustrative purposes, consider a system where two species co-exist in the analyte: protein monomers and protein aggregates of aggregation number N. It follows that protein monomers have the diffusion coefficient D_{mono}, while protein aggregates have the diffusion coefficient D_N. The Stokes-Einstein relation can be assumed to be applicable to the system, yielding the expression presented in Equation 7. In this expression, $R_{H,N}$ denotes the hydrodynamic radius of the species with aggregation number N ($N = 1$ to ∞) and $\eta$is the solvent viscosity.

$$D_N = \frac{k_BT}{6\pi\eta R_{H,N}} \tag{7}$$

Denoting the total number of monomers as n_{mono} and the number of N-mers as n_N, the total number of protein molecules becomes $n = n_{mono} + Nn_N$ and the total initial protein concentration $c_0 = c_{mono} + Nc_N$. Using the last part of Equation 5, an expression (Equation 8 for the total number of protein molecules in channel (ii) can be derived. Note that $c_{mono} + Nc_N = c_0$.

$$n_{(ii)}(t) = \frac{Ac_0}{2}\sqrt{Dt} \Rightarrow \Big/ n_{(ii)} = n_{(ii),mono} + Nn_{(ii),N} \Big/ \Rightarrow n_{(ii)}(t) = \frac{A}{2}\left(c_{mono}\sqrt{D_{mono}t} + Nc_N\sqrt{D_{mono,N}t}\right) \tag{8}$$

Due to the system consisting of two species in this example system, the diffusion coefficient and hydrodynamic radius (Equation 7) will present themselves as averages based on the concentration ratio $\frac{c_{(i)}}{c_{(ii)}}$. Combining the middle and last parts of Equation 6 and Equation 8, the average diffusion coefficient can be expressed as in Equation 9. Note that this corresponds to a mass weighted average of $\langle\sqrt{D}\rangle$.

$$\langle D\rangle = \frac{1}{t}\left(\frac{2an_{(ii)}}{c_0V}\right)^2 = \frac{1}{t}\left(\frac{c_{mono}\sqrt{D_{mono}t} + Nc_N\sqrt{D_{mono,N}t}}{c_0}\right)^2 = \left(\frac{c_{mono}\sqrt{D_{mono}} + Nc_N\sqrt{D_N}}{c_0}\right)^2 \tag{9}$$

The expressed presented in Equation 9 can be further generalized and extended to a broader size distribution $f(N)$, which is a function of the aggregation number. This generalized expression can be found in Equation 10 below.

$$D = \left(\sum_N \frac{Nc_N}{c_0}\sqrt{D_N}\right)^2 where c_0 = \sum_N Nc_N \tag{10}$$

Lastly, by using the Stokes-Einstein relation presented in Equation 7, a final expression for the average hydrodynamic radius is achieved (Equation 11).

$$\frac{1}{\langle R_H\rangle} = \left(\sum_N \frac{Nc_N}{c_0}\frac{1}{\sqrt{R_{H,N}}}\right)^2 \tag{11}$$

S6. Negative Stain Electron Microscopy

A set of images were collected from the negative stain electron microscopy experiment, which are shown in Figure 9(a)-(f). These images, along with the image presented in Figure 3(b) in the main article, were decoded and given to a project student not involved in the project, without giving any information on what the images depicted. The project student measured the diameter of 50 particles per image, and took between 3-9 measurement per particle using the software ImageJ. In total, this encompasses 350 particles and 1789 data points. The data points were in terms of diameter and to make the result more easily comparable to the radius values found through other methods, these values were converted to radius measurements as well. The average value for the radius was found to be approximately 7 nm.

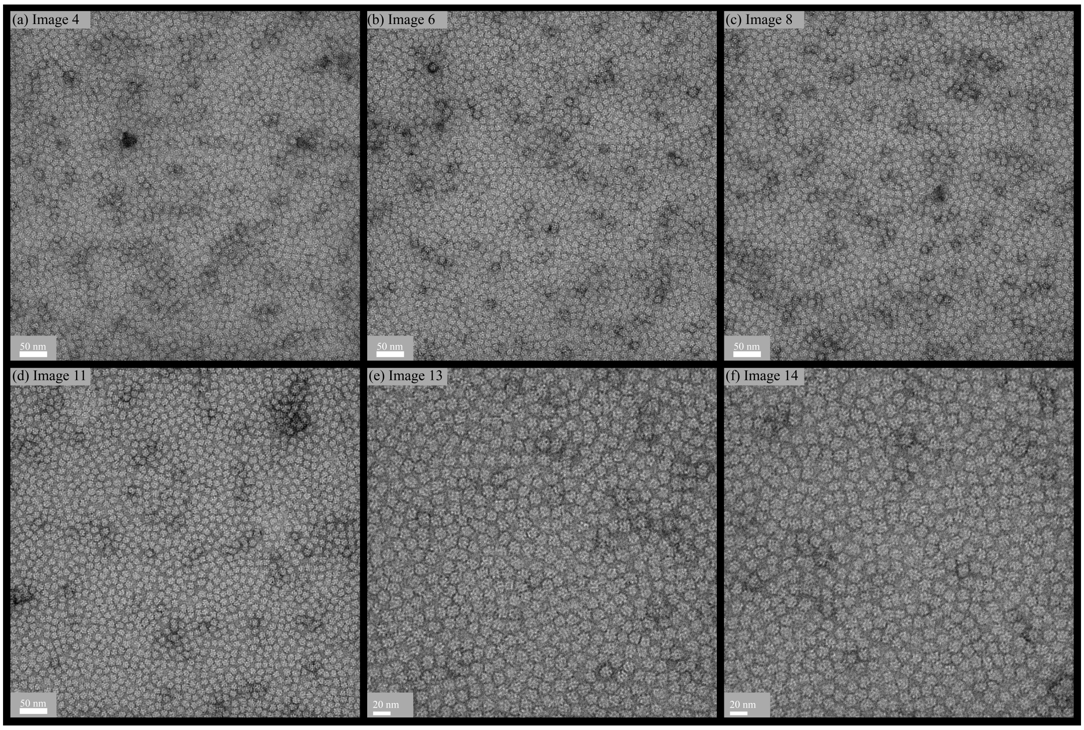

Figure 9: Additional negative stain images of 5 µM αB-crystallin at pH 8.0. Panels (a) through (f) show these images, where panels (e) and (f) are of a higher resolution.

As in the main article, panels (a) through (f) in Figure 9 indicate that the αBC assemblies appear to be spherical but also monodisperse in size. The histogram shown in Figure 9(g) displays some variation, but due to the experimental conditions during staining it is not possible to draw any conclusions regarding the variance in radius. During staining, the protein sample is dried onto a grid. It is unknown what orientation the protein oligomer assumes on the grid, and should it not be completely spherical different radius values may be measured. Furthermore, it is unknown exactly how the drying process affects the integrity of αBC assemblies. The assemblies could cluster, disintegrate, or a combination of both, which also would contribute to some variation of the measured radius. Another important aspect of negative stain imaging is naturally the stain - the stain is liquid at first but is then dried. It is not a given that the stain evenly distributes itself across the grid, which could affect the resolution of the image and thus also present difficulties in measuring the radius. The stain can in itself cause the protein to appear both smaller and bigger, based on the shape of the oligomers and the conditions in the grid preparation. The concentration is arguably too high as well, which could influence the ability to distinguish the edges of two αBC oligomers close together.

Despite the great uncertainty in the histogram regarding the distribution, the data is clearly centered about a radius value of approximately 6 nm. This corresponds very well to radius used in the homogeneous sphere model to fit the SAXS data, but also to the radius values obtained though dynamic light scattering (DLS) and MDS (approximately 7.4 nm and 7 nm, respectively). It can thus be said that the conditions in the preparation of grids for negative staining does not disrupt the oligomers completely. They appear to be intact despite the harsh conditions that αBC is exposed to.

References

(1) T. Nieuwkoop, B. R. Terlouw, K. G. Stevens, R. A. Scheltema, D. de Ridder, J. van der Oost and N. J. Claassens, *Nucleic Acids Res*, 2023, **51**, 2363–2376.
